# An effective field theory treatment of the production and annihilation of magnetic monopoles and their relic abundance

**DOI:** 10.1140/epjc/s10052-022-10864-2

**Published:** 2022-10-06

**Authors:** Luciano M. Abreu, Pedro C. S. Brandão, Marc de Montigny, Pierre-Philippe A. Ouimet

**Affiliations:** 1 Instituto de Física, Universidade Federal da Bahia, Campus Ondina, Salvador, Bahia 40170-115 Brazil; 2 Faculté Saint-Jean, University of Alberta, Edmonton, AB T6C 4G9 Canada; 3 Department of Physics, University of Regina, Regina, SK S4S 0A2 Canada

## Abstract

We revisit the thermal production and annihilation of magnetic monopoles and their relic abundance in order to gain a deeper physical interpretation on the monopole phenomenology predicted from the Baines et al.’s effective field theory, recently proposed in the description of monopole pair production via Drell–Yan and photon fusion processes. In this sense, we use of the vacuum cross sections for the Drell–Yan reactions derived within the mentioned framework to evaluate the cross section averaged over the thermal distribution associated to other particles that constitute the hot medium where the monopoles propagate. In the considered range of monopole mass with spin-zero and spin-half, our findings suggest that the thermally averaged cross sections for the pair production are highly suppressed, while at higher temperatures those for the annihilation of lighter pairs reach larger magnitudes. Besides, we observe that smaller temperature leads to a rate of annihilation for scalar monopoles smaller than the one for fermionic monopoles, which might be interpreted as a theoretical evidence of a more pronounced stability for spin-zero and heavier monopoles. Then we input these thermally averaged cross sections into the kinetic equation that describes the evolution of the monopole abundance via an extension of a freeze-out theory. Our results infer that heavier monopoles achieve the equilibrium at earlier stages of the expansion, and consequently at higher temperatures. In addition, larger monopole masses produce higher values of the relic abundance. Besides, the results indicate that the abundance does not behave differently for spin-zero and spin-half relic monopoles.

## Introduction

The existence of magnetic monopoles, first introduced by Dirac, offers an elegant explanation for the quantization of electric charge as well as being a feature of several beyond-the-Standard-Model theories. While these objects have been investigated theoretically in the past, no experimental evidence of their existence has yet been found [1–5].

With Run 3 of CERN’s LHC, expected later this year, about to push the energy frontier further, the possibility of direct detection of a magnetic monopole is once more in the cards. In addition to searches at ATLAS [6] the dedicated MoEDAL experiment will be taking data during this run. This experiment, located at the Interaction Point 8 on the LHC ring at CERN [7] has published some of the most recent bounds on monopole masses and charges. Other monopole searches include Refs. [8–13].

Recently, the analysis of the MoEDAL trapping detector provided mass limits in the range 1500–3750 GeV for magnetic charges up to 5$g_D$ for monopoles of spin 0, 1/2 and 1 [14]. A more recent search, for magnetic monopole production by the Schwinger mechanism in Pb–Pb heavy ion collisions at the LHC, excluded monopoles up to 75 GeV/$c^2$ to 70 GeV/$c^2$ for magnetic charges from 1$g_D$ to 3$g_D$, respectively, at a 95% confidence level [15]. That work exploited the idea that the production of monopole–antimonopole pairs are most probable within a strong magnetic fields created in heavy-ion collisions such as the high-energy Pb–Pb collision at the LHC [16]. Another active topic of research is the possibility of detection of bound states of monopole pairs, or monopolia [5, 17–22].

The new theoretical interest in monopoles, motivated by active experimental searches at higher energies, has stimulated new theoretical approaches to monopole physics. One significant theoretical challenge is the potential large value of the magnetic charge and, as a consequence, the non-perturbative nature of the corresponding field theory. In order to overcome this challenge, Baines et al. introduced an effective monopole theory with a velocity-dependent magnetic charge [23], in which slower monopoles would see a suppression of their interactions, thus leading to a perturbative theory for these monopoles. This effective theory is a U(1) gauge field theory which is motivated by using electric-magnetic dualisation. Baines et al. considered the production of point-like monopoles of spin 0, 1/2 and 1 via the photon-fusion and Drell–Yan processes [23].

The absence of observation of magnetic monopoles at the LHC up to this date, coupled with the non-observation of any relic monopoles argues that the relic monopole density is small. As a deeper investigation of Baines et al.’s effective theory, we will investigate the behavior of this description in the early universe. Along those lines, we examine the behavior of spin-zero and spin-half magnetic monopole pairs in a high-energy environment via the Drell–Yan process. Although contributions from photon fusion deserve attention, the approach we utilized for the thermally averaged cross section and the rate equation is well established for Drell–Yan-like reactions. Therefore, a treatment for the photon fusion would need a particular and complete development beyond the scope of this work. We plan to revisit this point in the near future.

An early discussion of monopole pair production from thermalized quark-gluon matter with the fireball model in heavy-ion collisions is in Refs. [24, 25]. More recently, the authors of Ref. [26] investigated lower bounds on the mass of magnetic monopoles in heavy-ions via the Schwinger process, relying on the rich production of monopoles afforded by strong magnetic fields and high temperatures. In Ref. [16], they also examined the Schwinger production of monopoles in peripheral heavy-ion collisions with the use of the wordline instanton method to all orders in the magnetic charge.

Since we are interested in the monopole freeze-out using the effective theory of Baines et al., in order to compute the relevant cross sections, we exploited results in Ref. [23]. In this paper, we obtain the thermal-averaged cross sections for monopole absorption, in analogy with recent work on the open flavour tetraquark state X(2900) in a hot hadronic medium Ref. [27, 28]. We obtained the thermally averaged cross section $\langle \sigma v_{rel}\rangle$ for the absorption of monopoles with an exact formula obtained by Cannoni for weakly interacting massive particles (WIMPs) and dark matter, and which is valid with an effective field theory where the masses of the annihilation products (here, the quarks) are much smaller than the monopole masses [29–31].

Equipped with the thermally averaged cross sections, we then turn to the monopole abundance by exploiting an approach utilized to describe the time evolution of the number density of weakly interacting massive particles (WIMP) candidates for dark matter following Refs. [32–34]. Therein, as in our present discussion of monopoles, when the Universe cools, the equilibrium abundance decreases until the annihilation reaction rate falls behind the Hubble expansion rate, where the interactions maintaining thermal equilibrium freeze out. As far as we know, this paper presents the first application of the formalism of Ref. [35] for the time evolution of the abundance of magnetic monopoles.

In Sect. 2, we briefly describe our effective Lagrangians for spin-zero and spin-half monopoles, for the production and annihilation of monopole–antimonopole pairs via the Drell–Yan processes, as well as the related cross sections. Then we compute the thermally-averaged cross sections for the production and annihilation of monopole–antimonopole pairs in Sect. 3. In both sections, we evaluate the effect of the velocity-dependent coupling and the magnetic moment contribution on the respective cross sections. The results of Sects. 2 and 3 allow us to investigate the relic abundance of monopoles, in terms of the monopole mass and the temperature, in Sect. 4. Among other findings, by comparing our results with a seminal 1982 paper by Turner, we observe some contrast regarding the dependence of the thermally averaged cross section on the temperature and the abundance’s dependence on $x=M/T$, and explain it in terms of the initial abundance considered in the rate equation. Lastly, we present concluding remarks in Sect. 5.

## The effective formalism

In order to investigate the production and annihilation mechanisms of monopoles required to assess the evolution of their abundance, we consider their electromagnetic interactions with ordinary photons. However, the fundamental theory of monopoles, if any, is not firmly established. In that regard, as in other studies, here we adopt the following ansatz: the use of effective field-theoretical models based on electric-magnetic duality. More concretely, we employ an effective *U*(1) gauge field theory introduced in Ref. [23] which describes the interaction of monopole fields with photons, by replacing the electric charge $q_e$ by the magnetic charge $g_D$. Accordingly, $g_D$ is quantized as required by Dirac’s quantisation rule, and provides the magnitude of the interaction of magnetically-charged fields with ordinary photons.

With these guiding principles, and keeping the monopole’s spin as a free parameter, we recall from Ref. [23] the effective Lagrangians for spin-zero monopoles $\phi$ and spin-half monopoles $\psi$, with the photon gauge field:1*M* is the monopole mass; $F_{\mu \nu }\equiv \partial _\mu A_\nu -\partial _\nu A_\mu$ and $D_\mu \equiv \partial _\mu -i g A_\mu$ are the field strength tensor and covariant derivative associated to the *U*(1) gauge field $A_\mu$ (i.e. the photon field).

As a first attempt, we devote our attention to the lowest-order diagrams contributing to the monopole pair production and suppression $q{\bar{q}}\rightarrow M{\bar{M}}$, i.e. Drell–Yan processes seen as monopole–antimonopole pair production from a quark–antiquark annihilation (and their respective inverse ones). These reactions have already been studied in Ref. [23], and we do not reproduce the details here, for the sake of conciseness. Notwithstanding, for completeness, we introduce some relevant quantities and information necessary to the subsequent sections. We remark that although this calculation is done with the quark–antiquark annihilation, it can be generalized for other fermionic fields of the standard model (like the electrons and positrons), by imposing the appropriate coupling describing the interaction of the fermions with the photon and adding a correction factor associated to the relevant internal degrees of freedom (i.e. for quarks they are related to the color and flavor states). We think that these should not display major qualitative changes compared to our findings reported below.

The spin-averaged cross section in the center of mass (CM) frame for the processes mentioned above is given by

2 $$\begin{aligned} \sigma _{ab \rightarrow cd} ^{(S)} (s) = \frac{1}{64 \pi ^{2} s} \frac{ |\vec {p}_{cd}| }{ |\vec {p}_{ab}|} \int d \Omega \overline{\sum _{S}} | {\mathcal {M}}_{ab \rightarrow cd}^{(S)} (s, \theta ) |^{2} , \end{aligned}$$

where $\sqrt{s}$ is the CM energy; $|\vec {p}_{ab}|$ and $|\vec {p}_{cd}|$ stand for the three-momenta of initial and final particles in the CM frame, respectively; ${\mathcal {M}}_{ab \rightarrow cd} (s, \theta )$ denotes the sum of the transition amplitudes of all processes contributing to the interaction; and the symbol ${\overline{\sum }}_{S}$ represents the sum over the spins (or polarizations and colors, as needed) of the particles in the initial and final state, weighted by the degeneracy factors $g_{1i}$ and $g_{2i}$ of the two particles forming the initial state.

Then, based on the effective Lagrangians introduced in Eq. (1), the amplitudes for the monopole pair production via Drell–Yan processes can then be calculated, and after some manipulations we get the total cross sections taking the limit of negligible quark (or other fermions) masses when compared to heavy monopoles [23]:

3 $$\begin{aligned} {\sigma ^{(S=0)}_{q{\bar{q}}\rightarrow M{\bar{M}}}(s)}= & {} \frac{5\pi \alpha _g\alpha _e}{27s}\beta ^3 \nonumber \\ {\sigma ^{(S=1/2)}_{q{\bar{q}}\rightarrow M{\bar{M}}}(s)}= & {} \frac{10\pi \beta \alpha _e\alpha _g}{27s}(3-\beta ^2), \end{aligned}$$

where $\alpha _e = e^2 /(4\pi )$ and $\alpha _g = g^2 /(4\pi )$ are the fine structure constants for the electric and the magnetic charges, respectively, and $\beta = \sqrt{1 - 4 M^2/s}$ is the monopole velocity.

**Fig. 1:**
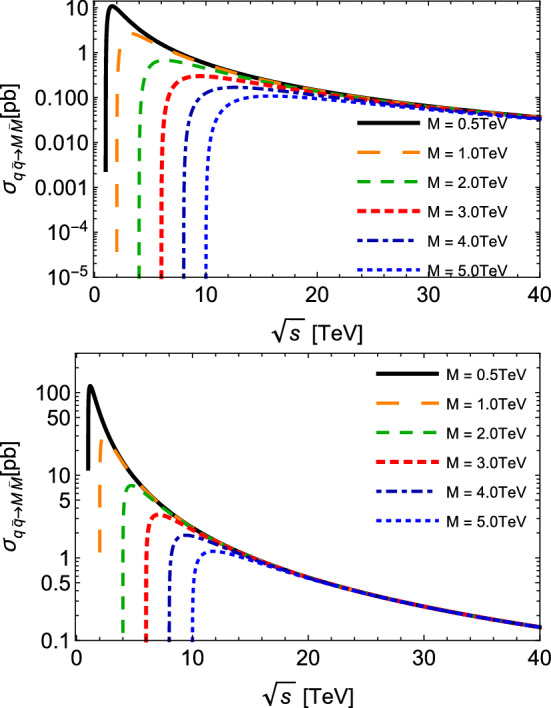
Total cross section for monopole–antimonopole pair production $q{\bar{q}}\rightarrow M{\bar{M}}$ as a function of the center-of-mass energy $\sqrt{s}$, with different values of the monopole mass *M*. Plots in the top and bottom panels describe spin-zero and spin-half monopoles, respectively

**Fig. 2:**
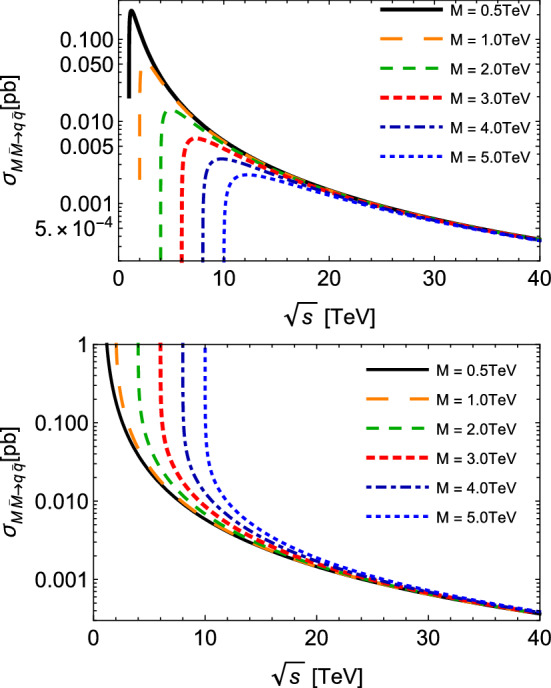
Total cross section for monopole–antimonopole pair annihilation $M{\bar{M}}\rightarrow q{\bar{q}}$ as a function of the center-of-mass energy $\sqrt{s}$, with different values of the monopole mass *M*. Plots in the top and bottom panels describe spin-zero and spin-half monopoles, respectively

The cross sections of the inverse processes, in which the monopole pair is annihilated via Drell–Yan processes, can be evaluated using the detailed balance relation, i.e.

4 $$\begin{aligned} {g}_{a}{g}_{b} |\vec {p}_{ab}|^2 {\sigma }_{a b \rightarrow c d } (s) = {g}_{c} {g}_{d} |\vec {p}_{cd}|^2 {\sigma }_{c d \rightarrow a b } (s). \end{aligned}$$

The total cross sections for the production and annihilation of monopole–antimonopole pairs are plotted in Figs. 1 and 2 as functions of the CM energy $\sqrt{s}$, for different values for the monopole mass between 0.5 and 5.0 TeV. The production cross sections are endothermic, and smaller values of *M* engender higher cross sections. From Figs. 1 and 2, we observe that the thresholds $\sqrt{s}_{min}$ increases with *M*, and that as the CM energy increases, the cross section $\sigma$ decreases for all processes, and that they trend towards the same behavior, almost indistinguishable at very high energies. At $\sqrt{s} \approx 15$ TeV, for example, this effective approach suggests cross sections with magnitudes of the order $\sim 10^{-1}$ pb and 1 pb for spin-zero and spin-half, respectively. If we look now at the inverse processes plotted in Fig. 2: due to their exothermic nature, they might have a different behavior near the thresholds. This is the case for spin-half monopoles: the curves for $\sigma _{inv} ^{(1/2)}$ become very large at their respective thresholds, and quickly decrease with larger $\sqrt{s}$. For $\sigma _{inv} ^{(0)}$, the use of Eq. (3) in (4) yields a linear dependence with the factor $\beta$ and therefore a distinct behavior. More interestingly, from moderate CM energies onward, these inverse cross sections become smaller than those for production reactions.

**Fig. 3:**
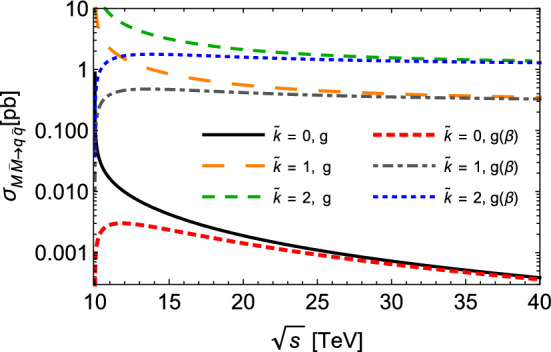
Total cross section for spin-half monopole–antimonopole pair annihilation $M{\bar{M}}\rightarrow q{\bar{q}}$ as a function of the center-of-mass energy $\sqrt{s}$, at the fixed value of the monopole mass $M=5$ TeV, but taking a velocity $( \beta )$ dependent coupling, $g ( \beta )$, and different values of the normalized parameter $\tilde{\kappa } = \kappa M$

For the sake of offering more perspectives and a deeper understanding on this effective approach, here we also include two more features in this analysis. The first one concerns the monopole’s velocity-dependent magnetic charge, implemented by the replacement $g \rightarrow g ( \beta ) = g \beta$. It is based on the electric-magnetic duality conjecture, which would lead to the similarity between the electron-monopole scattering cross section and the Rutherford formula by applying the above-mentioned replacement for $g(\beta )$. For details, see Ref. [23].

The second feature regards the insertion in the Lagrangian of spin-half monopoles (1) a magnetic moment generating term, given by [23]

5 $$\begin{aligned} {{\mathcal {L}}_{Mag.Mom.}^{(S=1/2)}}= & {} -\frac{i}{4} g ( \beta ) \kappa F_{\mu \nu } \overline{\psi } \left[ \gamma ^{\mu } ,\gamma ^{\nu } \right] \psi , \end{aligned}$$

where the parameter $\kappa$ encodes the magnitude of the magnetic moment. Obviously, with $\kappa = 0$ the original Lagrangian (1) is recovered. The motivation for this contribution comes from the lack of knowledge upon the origin of the monopole’s magnetic moment. In this sense, alternative sources to the anomalous quantum-level interactions should be taken into account.

The effect of these two features has been estimated and is shown in Fig. 3, where we plotted the total cross section for spin-half monopole–antimonopole pair annihilation as a function of $\sqrt{s}$ and at a fixed value of the monopole mass, but taking the velocity-dependent coupling $g ( \beta )$ and different values of the normalized parameter $\tilde{\kappa } = \kappa M$. As discussed in the subsequent sections, only the suppression reactions are relevant for our purposes; besides, the magnetic-moment generating term acts only in the case of spin-half monopole. Thus, for the sake of conciseness, we do not display further figures with production reactions and spin-zero monopoles including the effects under analysis. From Fig. 3 we see that the velocity-dependent coupling produces the lowest cross sections at smaller energies, but tends to the same behavior of the $\beta$-independent curve as the energy increases. Another interesting aspect is that $g ( \beta )$ engenders a modification near the threshold: the processes acquires an artificial exothermic nature. This is due to the additional $\beta ^2$ factor yielded in the cross section, which cancels a similar factor coming from the left-hand side in the detailed balance used to generate the annihilation cross section. In the end, ${\sigma ^{(S=1/2)}_{ M{\bar{M}}\rightarrow q{\bar{q}}}(s)}$ with $g ( \beta )$ has the same $\beta$-dependence as the ${\sigma ^{(S=1/2)}_{q{\bar{q}}\rightarrow M{\bar{M}}}(s)}$ with *g* in Eq. (3). On the other hand, the inclusion of the magnetic moment contribution causes a sizable augmentation of the cross section, which appears to have a strong dependence with the value of the parameter $\tilde{\kappa }$.

## The thermally averaged cross section for production and absorption of monopoles

Once the vacuum cross sections are obtained, the next step is to estimate the thermally averaged cross sections. Our interest in the present analysis stems from the mechanisms of monopole production and absorption from the early universe onward. In the initial stages of its formation, the correspondence between the time evolution *t* and the temperature *T* was described approximately by *t*(s) $\approx$ $T^{-2}$ (MeV) [36], which means a larger temperature as we move earlier in time. Keeping in mind that the temperature of the system determines the collision energy, the relevant dynamical quantity is the cross section averaged over the thermal distribution. It might be interpreted as the convolution of the vacuum cross section with thermal momentum distributions of the colliding particles. The thermal average acts suppresses part of the kinematical configurations close to the thresholds. In this sense, a strong threshold enhancement and the suppression observed in Figs. 1 and 2 might have no significance for our purposes.

Thus, let us define the cross section averaged over the thermal distribution for a reaction involving an initial two-particle state going into two final particles $ab \rightarrow cd$ as [29]

6 $$\begin{aligned} \langle \sigma _{a b \rightarrow c d } \, v_{a b}\rangle= & {} \frac{ \int d^{3} {\mathbf {p}}_a d^{3} {\mathbf {p}}_b \, f_a({\mathbf {p}}_a) \, f_b({\mathbf {p}}_b) \, \sigma _{a b \rightarrow c d } \,\,v_{a b} }{ \int d^{3} {\mathbf {p}}_a d^{3} {\mathbf {p}}_b \, f_a({\mathbf {p}}_a) \, f_b({\mathbf {p}}_b) } \nonumber \\= & {} \frac{1}{4 x_a ^2 K_{2}(x_a) x_b ^2 K_{2}(x_b) } \int _{z_0} ^{\infty } dz K_{1}(z) \,\, \nonumber \\&\times \sigma (s=z^2 T^2) \left[ z^2 - (x_a + x_b)^2 \right] \nonumber \\&\times \left[ z^2 - (x_a - x_b)^2 \right] , \end{aligned}$$

where $v_{ab}$ represents the relative velocity of the two initial interacting particles *a* and *b*; $\sigma _{ab \rightarrow cd}$ denotes the cross sections evaluated formerly for the different reactions shown in Figs. 1 and  2; the function $f_i({\mathbf {p}}_i)$ is the thermal distribution of particles of species *i*, which depends on the temperature *T*; $x _i = m_i / T$, $z_0 = max(x_a + x_b, x_c + x_d)$; and $K_1$ and $K_2$ the modified Bessel functions. Note that in the second line of Eq. (6), we applied the Boltzmann approximation, as in Ref. [29].

From Eq. (6), it can be presumed that for small values of $x_a, x_b$ the denominator reaches very high values; that is the case for finite temperatures and the relatively small masses of the quarks. Consequently, $\langle \sigma _{a b \rightarrow c d } \, v_{a b}\rangle$ acquires very small magnitudes for monopole production processes with respect to the suppression ones; within the accuracy used in our numerical calculations, they become zero in most of the range of temperature considered. For this reason, hereafter we shall only consider the latter reactions.

To estimate the thermally averaged cross section for absorption processes $M{\bar{M}}\rightarrow q{\bar{q}}$, it is more convenient to write it as a function of

7 $$\begin{aligned} x = M/T, \end{aligned}$$

and the vacuum cross section in terms of the dimensionless variable $y = s / 4M^2$ [30], and after some algebra, one finds:

8 $$\begin{aligned} \langle \sigma _{M{\bar{M}}\rightarrow q{\bar{q}}} \, v_{M{\bar{M}}}\rangle ^{(S=0)}= & {} \frac{20\pi \alpha _g\alpha _e x}{27 M^2 K_{2}^2(x)} \left[ I_{-\frac{1}{2}} - I_{-\frac{3}{2}} \right] , \nonumber \\ \langle \sigma _{M{\bar{M}}\rightarrow q{\bar{q}}} \, v_{M{\bar{M}}}\rangle ^{(S=1/2)}= & {} \frac{10\pi \alpha _g\alpha _e x}{27 M^2 K_{2}^2(x)} \left[ 2 I_{-\frac{1}{2}} + I_{-\frac{3}{2}} \right] , \end{aligned}$$

where the function $I_{p}$ is given by

9 $$\begin{aligned} I_{p}= & {} \int _{1} ^{\infty } dy \, (y-1) \, y^{p} \, K_{1}\left( 2 x \sqrt{y} \right) . \end{aligned}$$

This form therefore enables one to analyze the behavior of this relevant observable according to the ratio between the monopole mass and temperature of the medium.

**Fig. 4:**
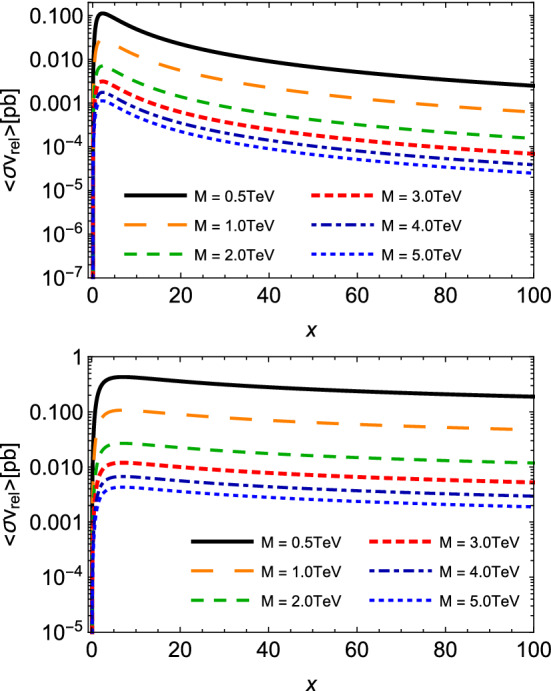
Thermally averaged cross sections for monopole pair annihilation $M{\bar{M}} \rightarrow q{\bar{q}}$ as a function of $x = M /T$, with different values of the monopole mass *M*. Plots in the top and bottom panels describe spin-zero and spin-half monopoles, respectively

In Fig. 4, we plot the thermally averaged cross sections of Eq. (8) as a function of the parameter *x* defined in Eq. (7). Here again, higher magnitudes are achieved for reactions involving monopoles with smaller masses, and as the temperature decreases (i.e. the variable *x* augments) this difference is kept nearly constant within the considered range of *x*. Also, the respective magnitudes for spin-zero are smaller than those for spin-half monopoles in the region of small *x*. We point out that, as we increase *x*, $\langle \sigma _{M{\bar{M}}\rightarrow q{\bar{q}}} \, v_{M{\bar{M}}}\rangle$ for spin-zero drops faster than that for spin-half; indeed, the latter suffers just a very slight decrease in the studied range. In other words, the decreasing of temperature engenders a rate of annihilation for scalar monopoles smaller than the one for fermionic monopoles. In the context of our effective approach, it might be interpreted as a theoretical evidence of a more pronounced stability for spin-zero and for heavier monopoles. It is noteworthy that the difference between the magnitudes of thermally averaged cross sections for the annihilation reactions and the production reactions might, in principle, play an important role in the search for monopoles in future heavy-ion colliders and in the evolution of the monopole abundance of cosmic origin. We will investigate the evolution of this abundance in the next section.

**Fig. 5:**
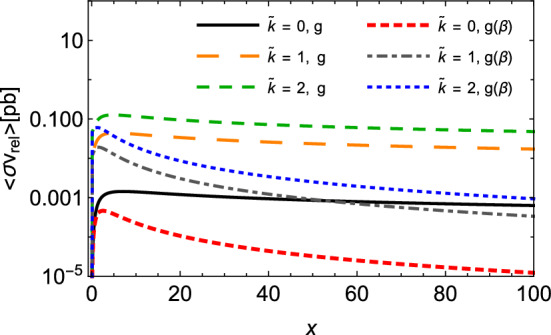
Thermally averaged cross section for spin-half monopole pair annihilation $M{\bar{M}} \rightarrow q{\bar{q}}$ as a function of $x = M /T$, at the fixed value of the monopole mass $M=5$ TeV, but taking a velocity $( \beta )$ dependent coupling, $g ( \beta )$, and different values of the normalized parameter $\tilde{\kappa } = \kappa M$

We finish this section by evaluating the effect of the velocity-dependent coupling $g ( \beta )$ and the magnetic moment contribution on the thermally averaged cross section for spin-half monopole pair annihilation $M{\bar{M}} \rightarrow q{\bar{q}}$. It is displayed in Fig. 5. It can be seen that the velocity-dependent coupling produces the lowest thermally averaged cross sections even at smaller values of *x*, and presents a more pronounced diminution as the temperature reduces. At higher values of *x*, the magnitude drops by a factor of almost two orders. On the contrary, the inclusion of the magnetic moment contribution generates a substantial growth of $\langle \sigma _{M{\bar{M}}\rightarrow q{\bar{q}}} \, v_{M{\bar{M}}}\rangle$, which might be of some orders of magnitude. Hence, these findings indicate non-negligible contributions coming from these two features, and may be relevant for the estimation of the evolution of monopole abundance.

## Relic abundance

### Framework

In this section, we estimate the evolution of the relic monopole abundance during the expansion of the universe. In general, one can use the thermally averaged cross sections estimated in the previous section as inputs in the momentum-integrated evolution equation abundance to assess the gain and loss terms, due to production and annihilation processes, respectively. Traditionally, various authors adopted some assumptions based on the so-called “freeze-out theory” [30, 34, 35, 37]. This framework has been employed in several scenarios, as in the analysis of dark matter. Its basis relies on the assumption that the evolution is divided into two stages: the first one is the early universe, during which expansion, particles (such as monopoles) become out of chemical equilibrium. The second stage is reached at a specific temperature, the “freeze-out temperature”, at which the production and annihilation rates of the stable particles become uniform. Past this stationary point, we are left with a residual number of particles, i.e. the relic abundance. For a given particle, the instant of the freeze-out depends on its mass and interactions; and it is commonly designated by $\tau _f$ (in our present case it can related to the freeze-out value $x_f$ of the variable *x*). From this perspective, the interactions of massive and stable monopoles with other particles might explain the monopole phenomenology. However, as it will be detailed hereafter, we adopt an improved version of the freeze-out theory, supposing that the actual abundance for a given monopole mass is known at an intermediate stationary point, which we designate as $x=\tilde{x}$ in order to distinguish it from the arbitrary point $x_f$ of the freeze-out approximation. The actual abundance at $\tilde{x}$ is used as the true initial condition for the differential equation driving its evolution (we refer the reader to Ref. [30, 35] for a more detailed discussion).

We begin with the rate equation governing the evolution of the relic number density *n* [34, 35]:

10 $$\begin{aligned} \frac{dn}{dt}+\frac{3{\dot{R}}}{R}n=-\left\langle \sigma _{M{\bar{M}}\rightarrow q{\bar{q}}}v_{M{\bar{M}}} \right\rangle (n^2 - n_0^2) , \end{aligned}$$

where $\left\langle \sigma _{ann} v \right\rangle$ is the thermally averaged cross section for the annihilation process (i.e. $M{\bar{M}}\rightarrow q{\bar{q}}$ in our case); $n_0$ is the number density in the thermal equilibrium; *R* is the cosmic scale factor related to the Hubble parameter *R* through $H ={\dot{R}}/R = \sqrt{8\pi G \rho /3}$, with $G=1/M^2_P$ being the cosmological constant ($M_p = 1.22\times 10^{16}$ TeV is the Planck mass) and $\rho =\pi ^2 g_{\rho }T^4 / 30$ the total energy density of the universe ($g_{\rho }$ counts the relativistic degrees of freedom contributing to the energy density).

Remembering that $\left\langle \sigma _{M{\bar{M}}\rightarrow q{\bar{q}}}v_{M{\bar{M}}}\right\rangle$ in Eq. (8) has been given as a function of the parameter *x* defined in Eq. (7), it is convenient to write Eq. (10) in a different form. Given the entropy density of the universe $s = (2\pi ^2/45)g_sT^3$, where $g_s$ refers to relativistic degrees of freedom associated to the the total entropy density [30, 34, 35, 37], then the hypothesis that the expansion proceeds adiabatically imposes the conservation of the total entropy per comoving volume $S=R^3s$. In consequence, the quantity $Y \equiv N/ S = n/s$ appears as an appropriate observable to be employed in the present analysis. We obtain it by dividing Eq. (10) by *S*. Thereafter, the variable is changed by employing the correspondence $d/dt\rightarrow Hxd/dx$, enabling one to rewrite the rate equation for the relic abundance as function of *x* in the form,

11 $$\begin{aligned} \frac{dY}{dx}=\frac{C}{x^2}\left\langle \sigma _{M{\bar{M}}\rightarrow q{\bar{q}}}v_{M{\bar{M}}}\right\rangle (Y^{2}_{0}-Y^2), \end{aligned}$$

where $Y_0 = 45/(4\pi ^4)(g_d/g_s)x^{2}K_{2}(x)$ is the initial equilibrium abundance ($g_d = 1$ or 2, for scalar or fermionic monopoles, respectively), and $C=\sqrt{\frac{\pi }{45}}M_{P}M\sqrt{g_{*}}$, with

12 $$\begin{aligned} \sqrt{g_{*}}= \frac{g_s}{\sqrt{g_{\rho }}} \left( 1+ \frac{T}{3}\frac{d(\ln g_s)}{dT} \right) \end{aligned}$$

depending on the relativistic degrees of freedom $g_{\rho }$ and $g_s$. As pointed out for example in Ref. [38], the degrees of freedom remain almost constant over a limited range of temperature. Consequently, it sounds reasonable to neglect their temperature dependence. Furthermore, $g_{\rho }$, and $g_s$ differ only when there are relativistic particles present that are not in thermodynamic equilibrium with the photons, which is not our situation here. Thus, we take $g_s = g_{\rho } = 100$, and $\sqrt{g_{*}} = \sqrt{g_{s}}$ [30].

At this point, let us briefly elucidate a crucial assumption that we utilized: as done in the literature (see Refs. [30, 35]), we conjecture that for a given monopole mass, our effective approach predicts the total annihilation cross section averaged over the thermal distribution, and therefore, it is possible to estimate the actual monopole abundance at a given stationary point $\tilde{x}$ , which is then interpreted as the true initial condition for the rate equation in Eq. (11). This enables one to get the asymptotic value for the abundance at large *x*. In other words, the true abundance becomes dependent on the point $\tilde{x}$. Thereby, the relic abundance *Y*(*x*) can be defined by the piecewise function

13 $$\begin{aligned} Y(x)={\left\{ \begin{array}{ll} Y_1(x) , \, &{}\quad x\le \tilde{x}, \\ Y_2(x) , \, &{}\quad x\ge \tilde{x}, \end{array}\right. } \end{aligned}$$

where the function $Y_1(x)$ describes the evolution before $\tilde{x}$, and $Y_2(x)$ is the actual abundance governed by the rate equation (11) for $x \ge \tilde{x}$, with the true initial condition $Y_2(\tilde{x}) = Y_1(\tilde{x})$. Hence, the differential evolution in temperature starts at $x = \tilde{x}$, where *Y*(*x*) is continuous and differentiable.

To determine the expression for the function $Y_1(x)$ and the condition for the stationary point $\tilde{x}$, we make use of the quantity $\Delta = Y - Y_0$, which represents the amount of abundance distant from equilibrium. The non-trivial stationary point is obtained from the condition $d \Delta / d x = 0$, which produces the quadratic equation

14 $$\begin{aligned} \Delta ^2 (\tilde{x} ) + 2 Y_0 (\tilde{x}) \Delta (\tilde{x} ) \frac{1}{ R (\tilde{x} )} \left. \frac{dY_0}{dx}\right| _{ \tilde{x}} = 0, \end{aligned}$$

where $R (x) = (C/x^2)\left\langle \sigma _{M{\bar{M}}\rightarrow q{\bar{q}}}v_{M{\bar{M}}}\right\rangle$. So, the physical non-negative solution of Eq. (14) is given by

15 $$\begin{aligned} \Delta (\tilde{x} ) = - Y_0 (\tilde{x}) + \sqrt{Y_0^2 (\tilde{x} ) - \frac{1}{ R (\tilde{x} )} \left. \frac{dY_0}{dx}\right| _{ \tilde{x}} }, \end{aligned}$$

which engenders the following actual abundance at $\tilde{x}$,

16 $$\begin{aligned} Y (\tilde{x}) = \sqrt{ Y_0^2 (\tilde{x} ) - \frac{1}{ R (\tilde{x} )} \left. \frac{dY_0}{dx}\right| _{ \tilde{x}} } . \end{aligned}$$

As a consequence, the matching point between the solutions in the two regions of *x* can be estimated by means of $\Delta (\tilde{x}) = \tilde{c} Y_0 (\tilde{x})$, where $\tilde{c}$ is a numerical factor determined by the expression

17 $$\begin{aligned} \tilde{c} = \sqrt{1 - \frac{1}{ Y_0^2 (\tilde{x} )R (\tilde{x} )} \left. \frac{dY_0}{dx}\right| _{ \tilde{x}} } - 1 . \end{aligned}$$

Hence, the well-defined form for $Y (\tilde{x})$ in Eq. (16) can be used to express the function $Y_1(x)$ as

18 $$\begin{aligned} Y_1(x) = \sqrt{Y^2_0 - \frac{1}{R (x)}\frac{dY_0}{dx}}, \end{aligned}$$

which can be rewritten in the form

19 $$\begin{aligned} Y_1(x) \equiv (1 + \delta (x) ) Y_0(x), \end{aligned}$$

where

20 $$\begin{aligned} \delta (x) = \sqrt{1 - \frac{1}{Y^2_0 (x) R (x)}\frac{dY_0}{dx}} - 1. \end{aligned}$$

Different values for $\delta (\tilde{x})$ have been proposed in the literature in distinct physical scenarios (see Ref. [35] for a detailed discussion). As a first attempt, here we adopt the condition $\Delta (\tilde{x}) = Y_0 (\tilde{x})$, yielding $Y_2 (\tilde{x}) = Y_1 (\tilde{x}) = 2 Y_0 (\tilde{x})$. We remark that a similar choice is employed in Refs. [33, 34] for the approximate initial condition at the freeze-out point in other contexts. As a result, this assumed condition produces $\delta (\tilde{x}) = 1$, which is equivalent to

21 $$\begin{aligned} - \left. \frac{1}{Y_0(\tilde{x})}\frac{dY_0}{dx} \right| _{ x = \tilde{x}} = \left[ 3\frac{C}{x^2}\left\langle \sigma _{M{\bar{M}}\rightarrow q{\bar{q}}}v_{M{\bar{M}}}\right\rangle Y_0 \right] _{ x = \tilde{x}}. \end{aligned}$$

Finally, it is also useful to analyze the chemical potential of monopoles. It is related to the “affinity”, defined as ${\mathcal {A}} = - \sum _i \nu _i \mu _i$, where $\mu _i$ are the chemical potentials of the species of type *i*, and $\nu _i$ are the stoichiometric coefficients, which are assumed to be negative for monopoles and antimonopoles, yielding ${\mathcal {A}} = 2 \mu$ [35]. If we assume that it is related to the *log* of the ratio between the production and annihilation rates, i.e. the two terms in the right-hand side of the rate equation in (10), then one can write the chemical potential as

22 $$\begin{aligned} \mu =\frac{M}{x}\ln {\left( \frac{Y}{Y_0} \right) }. \end{aligned}$$

### Numerical results and discussion

We can now estimate the relevant quantities introduced previously. Firstly, we find the magnitudes of $\tilde{x}$ required by the condition in Eq.  (21). They are shown in Tables 1 and  2 for different values of the spin-zero and spin-half monopole mass, respectively. We notice that $\tilde{x}$ decreases as *M* increases, due to the *M*-dependence of the thermally averaged cross section, *C* and $Y_0$, encoded in Eq. (21). This means that heavier monopoles reach the equilibrium at higher temperatures; that is, in older epochs. Let us also remark that, although in principle the dependence on the spin is present in the cross section and degenerescence factor $g_d$ in the condition (21), the numerical solutions for $\tilde{x}$ do not present sizable differences between spin-zero and spin-half, suggesting that they would have left a relic abundance at the same moment of evolution of the universe, viz. the same temperature $\tilde{T}$ engendered by the stationary point $\tilde{x}$. Given the rather small values of *M* we are treating, in the last array of Table 1 we also included a much higher mass, chosen accordingly to Refs. [5, 39] where that higher mass is related to the upper limit imposed by the nucleosynthesis constraints on the abundance of relic monopoles.

**Table 1:** Magnitudes of temperature $\tilde{T}$ and $\tilde{x} = m / \tilde{T}$ obtained from the condition in Eq. (21) for different values of the spin-zero monopole mass

Mass (TeV)	$\tilde{T}$ (TeV)	$\tilde{x}$
0.5	0.02	29.42
1.0	0.03	28.75
2.0	0.07	28.08
3.0	0.11	27.69
4.0	0.15	27.41
5.0	0.18	27.20
$10^4$	486.38	20.56

**Table 2:** Magnitudes of temperature $\tilde{T}$ and $\tilde{x} = m / \tilde{T}$ obtained from the condition in Eq. (21) for different values of the spin-half monopole mass

Mass (TeV)	$\tilde{T}$ (TeV)	$\tilde{x}$
0.5	0.02	29.82
1.0	0.03	29.15
2.0	0.07	28.49
3.0	0.11	28.09
4.0	0.14	27.82
5.0	0.18	27.60
$10^4$	492.61	20.30

**Fig. 6:**
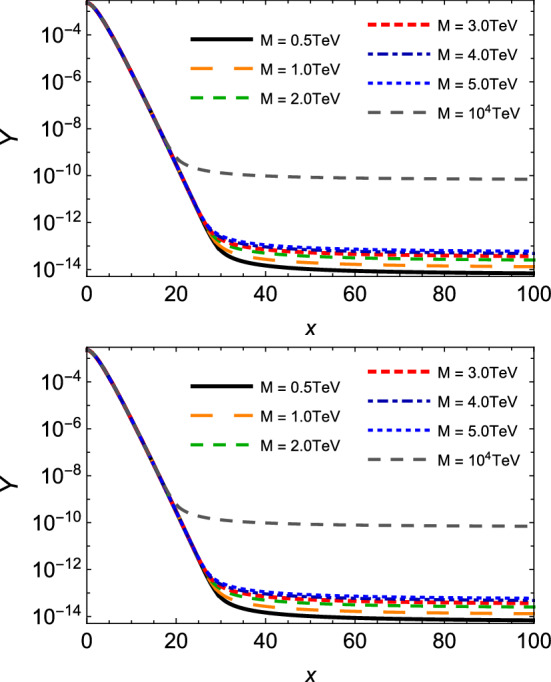
The relic abundance *Y*(*x*) for monopole as a function of $x = M /T$, according to Eq. (13), taking different values of the monopole mass *M*. Plots in the top and bottom panels: cases of spin-zero and spin-half monopoles, respectively

Figure 6 shows plots of the relic abundance *Y*(*x*) for spin-zero and spin-half monopoles as a function of $x = M /T$, according to the piecewise function Eq. (13), with different values of the monopole mass *M*. We notice that before the stationary point, the abundance evolves almost in the same way for different values of the monopole mass. From larger values of $\tilde{x}$, the curves become perceptibly distinct. Because heavier monopoles experience smaller stationary points, they achieve the equilibrium at earlier stages of the expansion (i.e. higher temperatures). No less important is that the increase of *M* produces higher values of the relic abundance. Specifically, an augmentation of one order of magnitude in *M* yields roughly a similar growth in the magnitude of *Y* at large *x*. Besides, the results suggest that the abundance does not behave differently for spin-zero and spin-half relic monopoles.

From the quantitative point of view, the values estimated for the relic abundance are clearly high, in view of the effective formalism and the magnitudes of the couplings and quantities taken. In that regard, the density of relic monopoles is naturally modified for different set of values for the relevant parameters, e.g. $g, \sqrt{g_{*}}, \sqrt{g_{s}}$ and so on. Notwithstanding, since the purpose here is not to make very accurate predictions, we postpone a more detailed analysis of this issue for future studies.

**Fig. 7:**
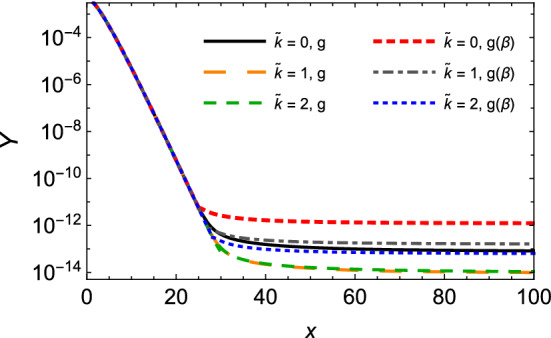
The relic abundance *Y*(*x*) for spin-half monopole as a function of $x = M /T$, according to Eq. (13), at the fixed value of the monopole mass $M=5$ TeV, but taking a velocity $( \beta )$ dependent coupling, $g ( \beta )$, and different values of the normalized parameter $\tilde{\kappa } = \kappa M$

Given the point raised in the previous paragraph, however, we have also performed an estimation of the effect of including the velocity dependent coupling $g(\beta )$ and the magnetic moment contribution on the relic abundance for the spin-half monopole. The are presented in Fig. 7. Interestingly, we have sizeable modifications with respect to the curves presented in Fig. 6. This can be understood as follows. The kinetic equation (11) that guides *Y*(*x*) for $x \ge \tilde{x}$ has in its right-hand side the magnitude of the gain and loss terms driven by the thermally averaged cross section. Furthermore, the variation of $\langle \sigma _{M{\bar{M}}\rightarrow q{\bar{q}}} \, v_{M{\bar{M}}}\rangle$ with $g ( \beta )$ and $\tilde{\kappa }$ is sufficient to alter the initial value $Y(\tilde{x})$ determined from Eq. (21). In particular, the velocity-dependent coupling produces the lowest thermally averaged cross sections, which by its turn gives higher $Y(\tilde{x})$, and therefore makes a faster stabilization of *Y*(*x*), since for higher *x* the $\langle \sigma _{M{\bar{M}}\rightarrow q{\bar{q}}} \, v_{M{\bar{M}}}\rangle$ with $g ( \beta )$ is even lower. Conversely, the inclusion of the magnetic moment contribution generates a substantial growth of $\langle \sigma _{M{\bar{M}}\rightarrow q{\bar{q}}} \, v_{M{\bar{M}}}\rangle$, a reduction of $Y(\tilde{x})$, and hence a slower stabilization of *Y*(*x*).

It is useful to compare our findings to others in the literature. For instance, in the seminal paper [40], the thermal production of magnetic monopoles has been calculated by using the monopole–antimonopole annihilation cross section and the detailed balance, and starting with an initial vanishing abundance. It has been found that the final abundance of monopoles depends upon $$x_i^3 e^{-2 x_i} $$, with $$x_i = M / T_t$$ ($$ M \sim 10^{13}$$ TeV and $$T_i$$ being the initial temperature of the production). Obviously, it is hard to perform a quantitative comparison because of the different purposes and values of the quantities considered (e.g. the mass used is very large with respect to the range considered here). But we remark some qualitative features of the approach employed in Ref. [40]: the thermally averaged cross section has a general dependence on the temperature as $$T^{-2}$$, and the relic abundance decreases monotonically for large *x*. These outcomes are clearly in contrast with those reported above. The main reason comes from the fact that the author of Ref. [40] uses only the solution of rate equation with a negligible initial abundance in the whole range of *x*, in some sense considering only the behavior of the function $$Y_1$$, in opposition to the piecewise function in Eq. (13).

**Fig. 8:**
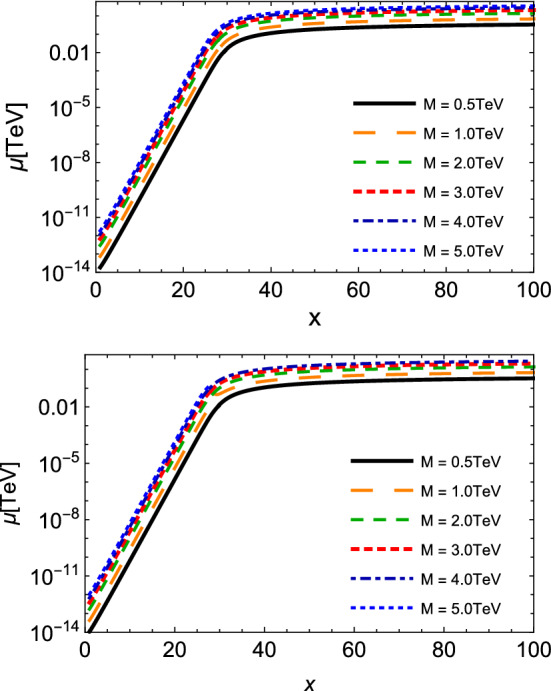
The chemical potential for monopoles as a function of $x = M /T$, according to Eqs. (22) and (13), taking different values of the monopole mass *M*. Plots in the top and bottom panels: cases of spin 0 and 1/2, respectively

We finish this analysis by showing in Fig. 8 the plots of the evolution of the chemical potential as a function of $x = M /T$ for spin-zero and spin-half monopoles, according to Eqs. (22) and (13), for different values of *M*. Since a non-zero chemical potential means a process out of chemical equilibrium, we see that at earlier stages of the universe, $\mu$ increases by several orders of magnitude, but as *x* increases and the rate in the definition of $\mu$ decreases, this yields a moderate growth. At larger *x*, i.e. smaller temperatures, this rate is very small, and $\mu$ saturates to an asymptotic value and the abundance tends to be constant. Interestingly, the asymptotic value is just the monopole mass, similarly to the findings in Ref. [35].

## Concluding remarks

In this work, we have revisited the thermal production and annihilation of monopoles and their relic abundance, exploiting the monopole phenomenology described by the effective field theory recently proposed in the description of monopole pair production via Drell–Yan and photon fusion processes [23]. To this end, we have used the vacuum cross sections for the Drell–Yan reactions derived within the mentioned framework to estimate the cross section averaged over the thermal distribution associated to other particles that constitute the hot medium where the monopoles propagate. In the considered range of monopole mass with spin zero and spin half, our results suggest that the thermally averaged cross sections for the pair production are highly suppressed, while at higher temperatures, these cross sections for the annihilation of lighter pairs reach larger values. Besides, for smaller temperatures, the rate of annihilation for scalar monopoles is smaller than for fermionic monopoles, which might be interpreted as a theoretical evidence of a more pronounced stability for spin-zero and heavier monopoles. This might be relevant in the search for monopoles in future heavy-ion colliders and of cosmic origin.

With our thermally averaged cross sections as inputs, we have also employed the effective approach of Ref. [23] to describe the evolution of the monopole abundance by extending a framework utilized previously for the number density of dark matter candidates [32–35]. From our results, we can infer that heavier monopoles experience smaller stationary points, achieving the equilibrium at earlier stages of the expansion, i.e. at higher temperatures. In addition, monopoles with larger mass produce higher values of the relic abundance. Specifically, an increase by one order of magnitude in *M* yields roughly a similar growth in the magnitude of *Y* at large *x*. Our results also suggest that the abundance does not behave differently for spin-zero and spin-half relic monopoles.

To conclude, we emphasize that our general objective was to study the implications of the effective field theory of Ref. [23] on some aspects of the monopole phenomenology. Clearly, more accurate analyses are needed to improve the present investigation such as, for example, the impact of our distinct assumptions on the values of the relevant parameters, the inclusion of other variables of interest (e.g. magnetic background), and so on. We postpone them for future discussions.

## Data Availability

This manuscript has no associated data or the data will not be deposited. [Authors’ comment: The theoretical nature of this manuscript implies that there is no data obtained by experiment. The numeric computations can be obtained from the authors upon request.]
